# Antioxidant Activity of Polyphenolic Plant Extracts

**DOI:** 10.3390/antiox9010019

**Published:** 2019-12-24

**Authors:** Dimitrios Stagos

**Affiliations:** Department of Biochemistry and Biotechnology, University of Thessaly, 41500 Biopolis, Greece; stagkos@med.uth.gr

Plant polyphenols are secondary metabolites characterized by one or more hydroxyl groups binding to one or more aromatic rings [1]. Several thousand polyphenolic molecules have been identified in higher plants, including edible ones. Plant polyphenols are divided into two major groups, flavonoids and non-flavonoids. Flavonoids can be divided into flavanols, flavonols, anthocyanidins, flavones, flavanones, and chalcones. Non-flavonoids include stilbene, phenolic acids, saponin, and tannins [1]. Among the important biological properties exhibited by plant polyphenols, their antioxidant activity has raised a great interest [1]. A number of studies have shown that plant polyphenols can be used as antioxidants against different oxidative stress-induced diseases [2,3].

This special issue consists of 25 articles related to isolation of polyphenols and/or polyphenolic extracts from different plants, assessment of their antioxidant activity, their prevention from oxidative stress-induced diseases, and their use as food additives.

Several studies have well established that oxidative stress plays a significant role in the manifestation and the health complication of diabetes mellitus [4]. Thus, antioxidant compounds such as plant polyphenols have been suggested that they may be used for the prevention and/or treatment of this disease afflicting millions of people worldwide [5]. Etsassala et al. [6] have reported for the first time that methanolic extract from *Salvia africana-lutea* is a rich source of terpenoids, especially abietane diterpenes (e.g., 19-acetoxy-12-methoxycarnosic acid, 3β-acetoxy-7α-methoxyrosmanol, 19-acetoxy-7α-methoxyrosmanol, 19-acetoxy-12-methoxy carnosol, safricana lactones A and B) and triterpenes (e.g., oleanolic and ursolic acids, 11,12-dehydroursolic acid lactone and β-amyrin). The methanolic extract exhibited in vitro strong antioxidant and antidiabetic properties [6]. Therefore, the authors suggested that extracts from *Salvia africana-lutea* may be used for both the prevention and/or amelioration of the symptoms of diabetes mellitus [6]. In another study, Dienaite et al. [7] showed that polyphenolic extracts from roots and leaves of *Paeonia officinalis* exhibited free radical scavenging activity and inhibited α-amylase suggesting their possible use as antidiabetic agents. The authors also performed UPLC-Q/TOF analysis supplemented with the on-line HPLC–DPPH•-scavenging method which revealed 20 gallic acid derivatives as radical scavenging compounds, which might account for the overall antioxidant potential of the extracts [7]. *Rubus idaeus* L. (raspberry) is another plant containing polyphenols combining antioxidant and antidiabetic properties [8]. Wu et al. [8] isolated extracts from leaves, fruit pulp, and seed of *R. idaeus* and identified their polyphenols by HR-HPLC-ESI-qTOF-MS/MS method. All the *R. idaeus’* extracts, especially those from leaves, exhibited strong antioxidant activity and inhibited the activity of the digestive enzymes α-glucosidase and α-amylase indicating antidiabetic properties [8]. The authors also used docking analysis in order to suggest possible mechanisms accounting for the digestive enzymes’ inhibition by *R. idaeus’* polyphenols [8]. Moreover, in a review article, Vlavcheski et al. [9] presented studies investigating the antidiabetic activity of hydroxytyrosol, a polyphenol found in high concentration especially in olive oil. The main conclusions of their review literature were that hydroxytyrosol exhibits insulin-like effects in several cell types and antidiabetic activity in vivo [9]. Moreover, hydroxytyrosol has been shown to exert protection against diabetes-induced oxidative stress [9]. Thus, hydroxytyrosol given its low toxicity may be used for the prevention and/or treatment of diabetes mellitus, although further studies are needed to investigate its bioavailability and to fully elucidate its mechanism of antidiabetic action [9].

As mentioned hydroxytyrosol is present in high concentration in olive oil which also contains other potent polyphenolic antioxidants such as oleuropein, tyrosol, coumaric acid, caffeic acid, vanillic acid, ferulic acid, kaempferol, and quercetin [10]. Aprile et al. [11] assessed for the first time the antioxidant activity and the total polyphenolic content of olive fruits from the olive tree “Cellina di Nardò” (CdN), one of the most widespread cultivars in Southern Italy. The results showed that the fully maturation is the best harvest time to obtain a table olive with a high content in polyhpenols [11]. It was also shown that different treatments that are necessary to remove the bitterness of the raw olive and to stabilize them to obtain edible table olives, cause a loss in phenolic substances which also results in a loss of antioxidant activity [11]. In addition, Martinez et al. [12] examined if hydroxytyrosol extract from olive fruits could be used as preservative for fish patties. Their results showed that hydroxytyrosol extract had strong antioxidant activity in vitro and antimicrobial against *Staphylococcus aureus* [12]. Thus, hydroxytyrosol extract may be used in the food industry for extending shelf life of fish products. Apart from olive fruits and olive oil, olive mill wastewaters (OMWWs), the byproducts of the olive oil production process, are rich in polyphenolic compounds [13]. However, these polyphenols along with other organic compounds account for the environmental problems caused by the OMWW’s discard [14]. Thus, the isolation of polyphenols from OMWW has been suggested as a way, on the one hand to reduce the environmental pollution around olive oil production industries, and on the other hand to produce polyphenolic extracts possessing strong antioxidant activity [13]. However, one of the main problems regarding the use of polyphenols as antioxidant supplements is their low availability [15]. In order to increase polyphenols’ bioavailability, their encapsulation in different organic carriers has been suggested [16]. Thus, my research group conducted a study to find out the optimal conditions for the encapsulation of a polyphenolic extract from OMWW in maltodextrin and whey protein [17]. The results demonstrated that both tested carriers were effective for the production of antioxidant powder using encapsulation by spray drying, although in different conditions. Specifically, inlet/outlet temperature did not seem to affect maltodextrin samples’ antioxidant activity, but whey protein samples showed better antioxidant activity at lower temperatures (within the temperature range used; 100–160 °C) [17]. In addition, the encapsulated OMWW extract exerted protection from DNA damage induced by ROS [17]. Furthermore, an OMWW extract (encapsulated or not) has been reported for the first time to increase antioxidant mechanisms such as glutathione levels in endothelial cells suggesting their possible use for protection from oxidative stress-induced pathologies associated with the cardiovascular system [17]. We also demonstrated in another study that polyphenolic extract from *Rosa canina* fruit possessed strong free radical scavenging activity, inhibited ROS-induced DNA damage and improved redox status in human endothelial cells by reducing ROS and increasing GSH levels [18].

In addition, in a clinical study, Chen et al. [19] also demonstrated that antioxidant polyphenols from almond skins may prevent oxidative stress induced cardiovascular disease. Specifically, they showed, in a placebo-controlled, three-way crossover trial with a 1-week washout period, that consumption of almond skin polyphenols increased the polyphenols catechin and naringenin, the ratio of reduced glutathione (GSH) to oxidized glutathione (GSSG) and the activity of the antioxidant enzyme glutathione peroxidase (GPx) in plasma [19]. Moreover, the consumption of almond skin polyphenols inhibited low density lipoprotein (LDL) oxidation which is an important etiological factor for cardiovascular diseases [19].

Apart from cardiovascular pathologies, another chronic disease associated with oxidative stress is Alzheimer’s disease [20]. Diaz et al. [21] assessed in vivo the protective effect of epicatechin against oxidative stress-induced damage to neurons. In particular, they demonstrated that epicatechin administration to rats injected with Aβ_25–35_ reduced neurotoxicity, oxidative stress, and inflammation in the hippocampus [21]. Moreover, epicatechin administration decreased the immunoreactivity to heat shock protein (HSP)-60, -70, and -90 and neuronal death in the Cornu Ammonis 1 (CA1) region of the hippocampus, which favors an improvement in the function of spatial memory [21].

Intestinal diseases have also been associated with oxidative stress [22]. Specifically, oxidative stress has been shown to cause a defective barrier function leading to intestinal pathologies [22]. Thus, Yang et al. [23] investigated the protective effects of Red-osier dogwood (*Cornus stolonifera* Michx.) polyphenolic extracts against hydrogen peroxide-induced damage in Caco-2 intestinal epithelial cells. The results showed that Red-osier dogwood extract’s treatment increased cell viability and decreased ROS through increased expression of antioxidant enzymes such as hemeoxygenase-1 (HO-1), superoxide dismutase (SOD), and glutathione peroxidase (GPx) in Caco-2 cells [23]. The expression of all these enzymes was probably due to the enhanced protein expression of the nuclear factor (erythroid-derived 2)-like 2 (Nrf-2), the most important transcription factor regulating antioxidant genes’ expression [23]. Red-osier dogwood extract was also shown to increase the transepithelial resistance (TEER) value through inhibition of disorganization of tight junction proteins such as zonula occludens-1 (ZO-1) and claudin-3 [23]. Finally, Red-osier dogwood extract decreased in Caco-2 cells markers (e.g., interleukin 8) of inflammation which plays important role in intestinal diseases [23]. In general, there is interdependence between oxidative stress and inflammation resulting in many chronic diseases [24]. Anti-inflammatory activity was also shown to be possessed by polyphenolic extracts from mulberry species [25]. In particular, Negro et al. [25] isolated polyphenolic extracts from Italian mulberry local varieties belonging to *Morus alba* and *Morus nigra* species. The *M. alba* and *M. nigra* extracts contained five main anthocyanin compounds as identified by HPLC/DAD/MS analysis [25]. The extracts from all the tested mulberry varieties exhibited in vitro strong free radical scavenging and inhibited cyclooxygenase (COX) activity (a marker of inflammation) [25]. It is not only the polyphenols that affect the gastrointestinal system, but also the gastrointestinal digestion may affect polyphenols activity [26]. For example, David et al. [26] used a simulated in vitro digestion model to investigate gastrointestinal digestion’s effects on the antioxidant capacity of Cornelian (*Cornus mas* L.) cherry fruit extract. The results showed that presence of three anthocyanins (i.e., cyanidin-3-*O*-galactoside, pelargonidin-3-*O*-glucoside, and pelargonidin-3-*O*-rutinoside) found in Cornelian cherry fruits, was not significantly affected by the gastric digestion [26]. However, intestinal digestion decreased the anthocyanin content and antioxidant activity of the fruit extract indicating that its polyphenolic content stability during gastrointestinal digestion should be taken into consideration for estimating its bioavailability [26].

In addition, tea is one of the most known plants for its prevention from chronic diseases [27]. This tea’s preventive activity is attributed mainly to the antioxidant properties of its polyphenolic content [27]. Although there have been several studies on tea’s polyphenols, there is a need for ongoing research, since there are many tea varieties cultivated in various regions having different soil and climatic conditions which affect tea’s chemical composition and consequently its bioactivities [27]. Tang et al. [28] isolated polyphenolic fractions from 30 Chinese teas belonging to six categories, namely green, black, oolong, dark, white, and yellow teas, identified their polyphenols by HPLC and assessed their antioxidant capacity. The results showed that yellow, and oolong teas exhibited greater antioxidant activity and had higher polyphenolic content (e.g., catechins like epicatechin, epigallocatechin, epicatechin gallate, and epigallocatechin gallate) compared to dark, black, and white teas, green, [28].

In general, the phytochemical and pharmacognostical studies of local plant varieties, especially those used as traditional medicinal plants, is considered currently important for drug and food supplement development [29]. Thus, Karabagias et al. [30] examined the antioxidant properties of *Lavandula stoechas* grown in Greece. *L. stoechas* has been used in the traditional medicine since ancient times [30]. Both methanolic and aqueous extracts of *L. stoechas* exhibited in vitro antioxidant activity, but the latter was the most potent [30]. The major polyphenols found in *L. stoechas* aqueous extract were caffeic acid, quercetin-*O*-glucoside, lutelin-*O*-glucuronide and rosmarinic acid [30]. Moreover, fifty volatile compounds belonging to alcohols, aldehydes, ketones, norisoprenoids, and numerous terpenoids were identified by using HS-SPME/GC-MS [30]. In another study, Blando et al. [31] assessed the antioxidant activity of polyphenolic extracts from cladodes of *Opuntia ficus-indica* (L.). In particular, *O. ficus-indica* cladodes extracts exhibited in vitro strong free radical scavenging activity and increased antioxidant activity in human erythrocytes [31]. The extracts also protected erythrocytes from ROS-induced hemolysis [31]. Moreover, the *O. ficus-indica* extracts exerted significant antimicrobial activity against *Staphylococcus aureus* biofilm formation [31]. The observed bioactivities of *O. ficus-indica* extracts were probably attributed to its polyphenolic content, especially to piscidic acid, eucomic acid, isorhamnetin derivatives, and rutin [31]. Moreover, Osman et al. [32] identified by GC-MS the polyphenols of *Dialium indum* L. fruit and assessed in vitro their antioxidant properties. *D. indum* is native to Southeast Asia and its fruit is edible [32]. The results showed that the *D. indum* fruit contains phenolics, amino acids, saccharides, fatty acids, sesquiterpene, polyols, and dicarboxylic acids [32]. Interestingly, it was also demonstrated for the first time that the exocarp of *D. indum* fruit contains thirteen phenolic antioxidants (i.e., vanillic acid, syringic acid, ferulic acid, isoferulic acid, sinapic acid, vanillin, syringic aldehyde, *p*-hydroxybenzaldehyde, coniferyl aldehyde, *p*-hydroxybenzoic acid, homovanillic acid, *p*-coumaric acid, and sinapic aldehyde) [32]. *Averrhoa bilimbi* is another plant that has been used in traditional medicine of Asian countries [33]. Ahmed et al. [33] investigated the antioxidant activity of polyphenolic extracts and fractions from *A. bilimbi* leaves. The results showed that *A. bilimbi* leaf extract had in vitro high free radical scavenging capacity, especially the n-butanol fraction [33]. Moreover, *A. bilimbi* leaf extracts inhibited xanthine oxidase, an enzyme involved in oxidative stress and metabolic disorders [33]. Finally, docking analysis indicated that 5,7,40-trihydroxy-6-(1-ethyl-4-hydroxyphenyl) flavone-8-glucoside (cucumerin A) and afzelechin 3-*O*-alpha-L-rhamnopyranoside are the possible compounds that are responsible for xanthine oxidase inhibition [33]. 

Currently, there is a great interest for natural preservatives of foods instead of synthetic, since the latter present often harmful effects on human health [34]. The dosage of antioxidant compounds used as food preservatives should be regulated and the functionality should be evaluated to ensure stability [34]. For example, polyphenolic extracts from *Rosmarinus officinalis* L. (rosemary) has been widely used in food industry as antioxidant additive [35]. In some countries, the addition of rosemary extract as food additive is permitted, while in other countries it is not allowed [35]. Thus, Choi et al. [35] developed a quantitative high-performance liquid chromatography-photodiode array (HPLC-PDA) method for the determination of the amount of rosemary extract in various food products (e.g., edible oils, processed meat products and dressings). Moreover, the method could evaluate the antioxidant activity of rosemary extract in foods, and consequently its functional stability [35]. Thus, they found that in terms of antioxidant activity carnosic acid of rosemary extract is more stable than carnosol [35]. Fruit vinegars containing polyphenolic antioxidants are also used widely in food industry as condiments [36]. Liu et al. [37] assessed in 23 fruit vinegars their phenolic components by HPLC coupled with photometric diode array detector (HPLC-PDA), and their antioxidant activity in vitro. The most common polyphenols in fruit vinegars were gallic acid, protocatechuic acid, chlorogenic acid, caffeic acid, and *p*-coumaric acid [37]. Among the 23 tested vinegars, the most potent were balsamic vinegar of Modena (Galletti), Aceto Balsamico di Modena (Monari Federzoni), red wine vinegar (Kühne), and red wine vinegar (Galletti) [37]. 

Since, polyphenols are used as food additives, it is also interesting to find out how cooking affects their antioxidant activity. Gunathilake et al. [38] examined the effect of cooking (boiling, steaming, and frying) on the antioxidant activity of polyphenols from edible leaves of six species, *Centella asiatica, Cassia auriculata, Gymnema lactiferum, Olax zeylanica, Sesbania granadiflora,* and *Passiflora edulis*. The findings demonstrated that frying decreased polyphenols, flavonoids, carotenoids, and their antioxidant activities in all leafy vegetables [38]. The effects of boiling and steaming on polyphenols, carotenoids, and their antioxidant properties, varied according to the leaf type [38]. Thus, the results of the study are useful for choosing the appropriate cooking method of leafy vegetables for their antioxidant properties to be maintained.

The use of polyphenols as food additives has raised concerns about the safety for human health of extraction methods using organic solvents [39]. Thus, “green extraction methods” are being developed using modern technology, where less or no organic solvents are used to minimize environmental and health impacts. The most common of such “green methods” are microwave assisted (MAE), ultrasound assisted (UAE), pulsed electric field assisted, and enzyme assisted extractions, infrared irradiation (IR), pressurized liquid and supercritical fluid extractions [39]. Gajic et al. [40] investigated the optimal conditions for isolating polyphenols from black locust (*Robiniae pseudoacaciae*) flowers using UAE. The results showed that extraction time had the greatest influence on the total polyphenolic content, followed by the extraction temperature and ethanol concentration [40]. The optimal extraction conditions were 60% (*v/v*) ethanol, 59 °C, and 30 min at the liquid-to-solid ratio of 10 cm^3^ g^−1^ [40]. Moreover, the UAE gave a higher yield of polyphenols than the maceration and Soxhlet extraction [40]. The main polyphenols found in the extract were rutin, epigallocatechin, ferulic acid, and quercetin [40]. Bouaoudia-Madi et al. [41] have also used UAE to isolate polyphenolic extracts from *Myrtus communis* L. pericarp, a plant native of the Mediterranean basin. The results demonstrated that ethanol concentration, irradiation time, liquid solvent-to-solid ratio and amplitude affected significantly the yield of total polyphenolic content [41]. The optimal conditions for the isolation of *M. communis* polyphenolic extract using UAE were 70% *v/v* ethanol concentration, 7.5 min irradiation time and 30% liquid solvent-to-solid ratio [41]. In addition, isolation of *M. communis* polyphenolic extract using UAE was more efficient than MAE and conventional solvent extraction methods [41]. In another study, Quiroz et al. [42] used MAE to isolate polyphenolic extract from annatto (*Bixa orellana* L.) seeds. The results showed that the optimal extraction conditions were pH 7.0, solvent concentration 96% *v/v*, solvent-to-seed ratio 6:1 and microwave time 5 min [42]. The main polyphenols identified in the *B. orellana* seed extract were apigenin, hypolaetin, and caffeic acid derivatives [42]. Moreover, the *B. orellana* seed extract isolated by MAE had higher antioxidant activity and polyphenolic yield than that isolated by leaching [42]. Another “green extraction method,” far IF, was used by Azad et al. [43] in order to isolate antioxidant polyphenolic compounds from *Angelica gigas* Nakai. The results indicated that the optimal conditions for the IF polyphenolic extraction from *A. gigas* Nakai were at 220 °C for 30 min [43]. In addition, HPLC analysis showed that the main polyphenols found in the *A. gigas* Nakai extract were decursin and decursinol angelate [43]. 
